# Engineering Cyclodextrin Clicked Chiral Stationary Phase for High-Efficiency Enantiomer Separation

**DOI:** 10.1038/srep11523

**Published:** 2015-08-03

**Authors:** Jian Tang, Shapopeng Zhang, Yuzhou Lin, Jie Zhou, Limin Pang, Xuemei Nie, Baojing Zhou, Weihua Tang

**Affiliations:** 1 Key Laboratory of Soft Chemistry and Functional Materials (Ministry of Education), Nanjing University of Science and Technology, Nanjing 210094, People’s Republic of China

## Abstract

The separation of racemic molecules is of crucial significance not only for fundamental research but also for technical application. Enantiomers remain challenging to be separated owing to their identical physical and chemical properties in achiral environments. Chromatographic techniques employing chiral stationary phases (CSPs) have been developed as powerful tools for the chiral analysis and preparation of pure enantiomers, most of which are of biological and pharmaceutical interests. Here we report our efforts in developing high-performance phenylcarbamated cyclodextrin (CD) clicked CSPs. Insights on the impact of CD functionalities in structure design are provided. High-efficiency enantioseparation of a range of aryl alcohols and flavanoids with resolution values (*R*_*s*_) over 10 were demonstrated by per(3-chloro-4-methyl)phenylcarbamated CD clicked CSP. Comparison study and molecular simulations suggest the improved enantioselectivity was attributed to higher interactions energy difference between the complexes of enantiomers and CSPs with phenylcarbamated CD bearing 3-chloro and 4-methyl functionalities.

One of the essential attributes in nature is chirality. The existence of stereoisomers for a great variety of substances is a critical issue to be addressed, especially for those molecules of biological and pharmaceutical interests. The importance of chirality determination and chiral resolution of racemates has thus been emphasized, leading to the extensive research efforts devoted for chiral separation^1^. Chromatographic techniques and other resolution methods have been developed rapidly. High performance liquid chromatography (HPLC) coupled with chiral stationary phases (CSPs) has become as a robust technology for both analytical and preparative purposes^2^. The library of CSPs spans from Pirkle types to chiral crown ethers, macrocyclic glycopeptides antibiotics, ligand exchange, polysaccharides, proteins and cyclodextrins (CDs)^3^,^4^,^5^,^6^,^7^,^8^,^9^,^10^,^11^. CDs derived CSPs are advantageous for enantioseparation due to the versatile chemical modification to construct π-π interactions, dipole-dipole, ion-pairing, hydrogen bonding, electrostatic and steric repulsion interactions besides inclusion complexation to promote the chiral recognition process^12^,^13^. Moreover, CD CSPs are especially attractive for their compatibility and durability to be operated under different separation modes such as normal phase, reversed phase, polar organic and hydrophilic interaction modes. The manner of CD immobilization onto silica support contributes to mechanical properties and chiral selectivity of CSPs^14^. Click chemistry has estabilished as a facile method for the selective immobilization of functional molecules onto substrate surfaces under mild reaction conditions^15^. Click preparation of separation materials has thus generated a large spectrum of stationary phases for liquid chromatography^16^.

Click saccharides was first developed via Cu(I)-catalyzed azide-alkyne cycloaddition for the separation of polar compounds in hydrophilic interaction LC^17^. The enantioseparation of flavanone and selected racemates was further achieved with native β-CD clicked CSP in different mode HPLC^18^,^19^. Relatively low resolutions were observed due to the lack of π-π interactions. The development of both native and functionalized CDs clicked CSP was also explored in Ng’s group^20^,^21^,^22^. The native CD clicked CSP (CCN-CSP) afforded good enantioselectivities towards dansyl amino acids and flavonoids^20^. The comparison study of perphenylcarbamated β-CD CSP (CCP-CSP) and permethylated β-CD one revealed their enantioselectivities strongly depended on a complex interplay of ‘host–guest’ inclusion, hydrogen bonding, π–π and hydrophobic interactions^21^. Heptakis(6-deoxy-6-azido)-β-CD and heptakis(6-deoxy-6-azido-phenylcarbamoylated)-β-CD clicked CSPs were found to show better resolution for selected racemates than the mono-clicked counterpart^22^. Our recent studies found the surface loading of clicked CDs and column efficiency could also significantly improve the enantioselectivities of both native and perphenylcarbamated CD clicked CSPs^12^,^23^.

Except for CD modification and multiple clicking on the development of CD clicked CSPs, the impact of functionalities on phenyl group of perphenylcarbamated CDs has seldom been explored on the enantioselectivity of CD clicked CSPs so far. As known, the introduction of an electron-donating methyl group or an electron-withdrawing halogen at the 3- and/or 4-position of the phenyl ring on phenylcarbamated polysaccharide (cellulose and amylose) CSPs was found to improve their chiral recognition ability towards many racemates^11^,^24^,^25^. The nature and position of the substituents on the phenyl ring was explored with dichroro, dimethyl and chloromethylphenylcarbamate CDs CSPs, where the perphenylcarbamate CDs were immobilized onto silica at 2,3-positions or even 6-position of CDs using hexamethylene diisocyanate or 4,4’-diphenylmethane diisocyanate (corresponding to 15–20% of hydroxyl groups of the CD) as spacer^26^. All CSPs showed rather low enantiomeric resolving abilities, probably due to uncontrolled immobilization and low conlumn efficiency. NMR studies have, however, shown the interactions on the outer surface of chemically-modified CDs played an important role in the chiral recognition process^27^. It is thus noted that fine-tuned substitution of phenylcarbamate CD and well-controlled immobilization is the key to achieve high enantioselectivity. The preparation of perphenylcarbamate CD CSPs via mono-click approach is attractive to resolve the above-mentioned key challenges. The functionalities on CD rims are crucial to create the driving forces efficiently and construct largest interaction energy between the two complexes of CSP and two enantiomers to discriminate the enantiomers in the inclusion process.

To elucidate the impact of functionalities on CD rims on the chiral recognition ability of CD clicked CSPs, chloro and methyl substituents are introduced onto the 3 and 4 positions of phenyl groups on phenylcarbamated CD. Here we report the click synthesis of two structurally-defined CD clicked CSPs, i.e., per(3-chloro-4-methyl)phenylcarbamated CD clicked CSP (CCC3M4-CSP) and per(3-methyl–4-chloro)phenylcarbamated CD clicked CSP (CCM3C4-CSP) (Fig. 1). Their excellent enantioselectivities towards a range of racemates are explored in reversed-phase HPLC. A joint correlation study of enantioseparation, infrared spectroscopy and molecular simulation is conducted to provide microscopic insights into the significantly improved enantioseparation as observed. The large interaction energy difference between complexes of CCC3M4-CSP and enantiomers is attributed to its excellent chiral resolution ability.

## Results

### Synthesis and characterization of the CD clicked CSP

**Figure 2:**
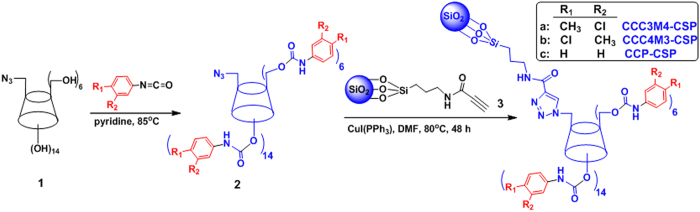
Click synthesis of perphenylcarbamated CD mono-clicked CSPs.

The click preparation of CD CSP was conducted according to the reported protocol^22^. Starting from mono-6^A^-azido-β-CD **1**^28^, mono-6^A^-azido-per(3-chloro-4-methyl)phenylcarbamated β-CD **2a** (or mono-6^A^-azido-per(3-methyl-4-chloro)phenylcarbamated β-CD **2b**) was prepared as a key intermdiate compound, which was further immobilized onto alkynyl functionalized silica gel **3**^22^ via “click” chemistry to afford the CCC3M4-CSP (or CCM3C4-CSP) over 90% yield (Fig. 2). CCP-CSP was also prepared as reference^12^.

**Figure 3:**
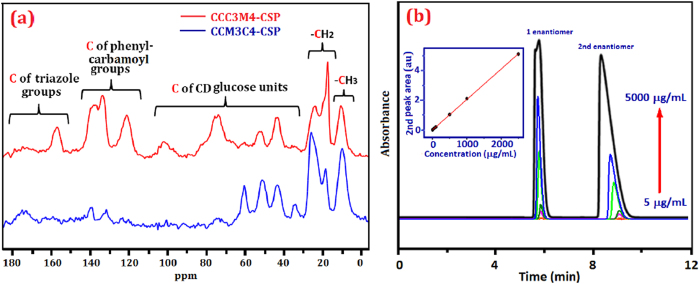
(**a**) Solid state ^13^C NMR spectra of CCC3M4-CSP and CCM3C4-CSP, (**b**) the enantioseparation chromatograms of flavanone with increased concentrations at CCC3M4-CSP with 1.0 mL/min methanol as mobile phase. The inset showing the standard curve of quantitative analyses for flavanone.

The successful click immobilization of CD was confirmed by solid state ^13^C NMR (Fig. 3a) and elemental analysis. Peaks between 140 and 110 ppm for the triazole groups indicate the reliable click of phenylcarbamate CD onto silica support. Signals between 140 and 110 confirm the successful functionalities of phenylcarbamate groups on CD. Signals in the region of 110-30 ppm are assigned to the carbon atoms in CD. The signals at 30-20 ppm are assigned to the C atoms of alkyl linkage between silica and triazole rings in CSPs. Peaks around 10 ppm are assigned to the C atoms of 4-methyl group in (3-chloro-4-methyl)phenylcarbamoyl groups. CCC3M4-CSP showed a content of 26.90% for carbon atom (C%), 1.546% for nitrogen atom (N%) and 4.317% for hydrogen atom (H%). And CCM3C4-CSP possessed 12.28% C, 0.822% N and 2.064% H. Both CSPs exhibited greatly increased contents of C, N, H atoms when compared with the corresponding C% (6.90%), N% (0.271%) and H% (1.526%) of alkynyl silica. The surface loading of CCC3M4-CSP and CCM3C4-CSP was calculated to be 0.37 and 0.18 μmol/m^2^ with [C%/(12 × N_C_ × S_silica_)] × 10^6^, where C% derived from elemental analysis of carbon, N_C_ is the carbon atom number per CD molecule (here N_C_ = 202) and S_silica_ is the surface area of silica gel (300 g/cm^2^)^12^. The much lower suface loading of CCM3C4-CSP than CCC3M4-CSP is consistent with the solid NMR results. The as-packed columns with CCC3M4-CSP and CCM3C4-CSP delivered a column efficiency up to 13142 and 12794 plates/m, respectively, determined using toluene as test compound at 1.0 mL/min MeOH. These column efficiencies are much higher than the literature reported CCN-CSP and CCP-CSPs^12^,^20^,^21^,^23^.

The quantitative analysis of CCC3M4-CSP was studied with both achiral separation of naphthalene and chiral separation of flavanone. Good linear relationship (R^2^ = 0.99975) was observed for the peak area of naphthalene with its concentration in the range of 50–500 μg/mL. And the linear fit for flavanone (R^2^ = 0.99985) was found within the concentration range of 5–2500 μg/mL (inset of Fig. 3b). The good repeatability of the separation for naphthalene (240 μg/mL) and flavanone (100 μg/mL) in six injections was demonstrated with low relative standard deviation (RSD) of 0.47% and 0.23%, respectively. The enantioseparation chromatograms of flavanone with increased concentrations with CCC3M4-CSP at 1.0 mL/min MeOH are depicted as Fig. 3b. Both the peak area and peak height are found to increase with increased concentration of flavanone. The resolution and the retention time of two enantiomers almost remained constant in low sample concentrations (ca ≤ 100 μg/mL). However, when analyte concentration was increased to higher than 500 μg/mL, the retention time of first peak remained constant but the retention time of second peak was declined, leading a decreased resolution. Distortion of the enantiomer peak was observed at high flavanone concentration as 5000 μg/mL. Such high sample loading demostrated the robustness of clicked column for enantioseparation.

### Enantioseparation performance

The functionalities-tuned enantioselectivities of phenylcarbamated CD clicked CSPs were investigated using 19 racemates including aryl alcohols, flavanoids and β-blockers as model analytes. Representative enantioseparation results for 19 racemates at their optimized separation conditions with two functionalized phenylcarbamated CD clicked CSPs are compared with the reference CCP-CSP. As shown in Table 1. The functionalities on phenyl group of perphenylcarbamated CDs are found to play great role on the enantioselectivities of the resulted CSPs. Among the three CSPs studied, CCC3M4-CSP exhibited the best enantioselectivities (including both α and *R*_*s*_) for 18 racemates except 1-*p*-tolybut-3-en-1-ol. And the peak resolutions (*R*_*s*_: 4.8 ~ 14.7) for most racemates with CCC3M4-CSP are at least one-fold higher than those obtained with the other two CSPs. Their enantioselectivities of CSPs are greatly related to the structures of racemates. It is noted that CCC3M4-CSP showed relatively improved resolution ability than CCP-CSP for racemates including 1-(4-flurophenyl)ethanol, 1-*p*-tolybut-3-en-1-ol, (*E*)-1,3-diphenylprop-2-en-1-ol, benzoin, 7-hydroxylflavanone, 4-hydroxylflavanone, hesperetin and naringenin on one hand. On the other hand, CCP-CSP exhibited significantly increased enantioselectivities than CCM3C4-CSP towards 1-(4-chlorophenyl)ethanol, 1-(4-bromophenyl)ethanol, 1-(4-chlorophenyl)but-3-en-1-ol, flavanone, 7-methoxyflavanone, 6-methoxyflavanone and clenbuterol.

CCC3M4-CSP showed excellent enantioselectivities for neutral racemates (including aryl alcohols and benzoin), flavanoids and basic racemates (ca. clenbuterol and isoprenaline), which possess a chiral center on α–position (ca. aryl alcohol and benzoin) or β-position (flavanoids) adjacent to aromatic rings. For analytes (ca. atenolol and propranolol) containing a chiral center on γ-position, only partial resolution was achieved with CCC3M4-CSP. The enantioseparation of aryl alcohols and flavonoids with CCC3M4-CSP was found to be strongly dependent upon their structures. For 1-phenylethanols, the introduction of an electron-withdrawing group to the *para*-position of phenyl group led to much improved resolutions and stronger retention with CCC3M4-CSP. And the preferential inclusion order of organohalides with β-CD was bromide>chloride>fluoride^29^, which corresponded to the *R*_*s*_ and retention time. A lose look at the enantioseparation data of 1-*p*-tolybut-3-en-1-ol and 1-(4-chlorophenyl)but-3-en-1-ol, we could find that the introduction of an electron-withdrawing group on *para*-position of aromatic ring also resulted in enhanced *R*_*s*_. This can be explained as stronger interactions constructed between CCC3M4-CSP with appropriate enantiomers via hydrogen-bonding, resulting in higher enantioselectivities^30^.

Comparing the resolutions of 1-(4-chlorophenyl)ethanol and 1-(4-chlorophenyl)but-3-en-1-ol, one would find that a double bond incorporation adjacent to the chiral center (C atom) of aryl alcohol could greatly increase the resolution, mainly due to the formation of π–π interactions between C = C bond and carbamate groups of CSP. Comparison with CCN-CSP and CCP-CSP^20^,^21^,^12^, CCC3M4-CSP afforded much higher resolution for aryl alcohols. Impressively, the *R*_*s*_ of (*E*)-1,3-diphenylprop-2-en-1-ol was as high as 13.7, which was over 3 and 11 times higher than that obtained with mono-6-deoxyphenylimine-β-CD and mono-6-deoxybenzimide-β-CD, respectively^31^. These behavious can be explained with the enhanced π-π conjugation between C=C bond and phenyl group via forming stronger π–π interactions, dipole-dipole and steric repulsion interactions with the existence of 3-chloro and 4-methyl in phenylcarbamate moieties.

**Figure 4:**
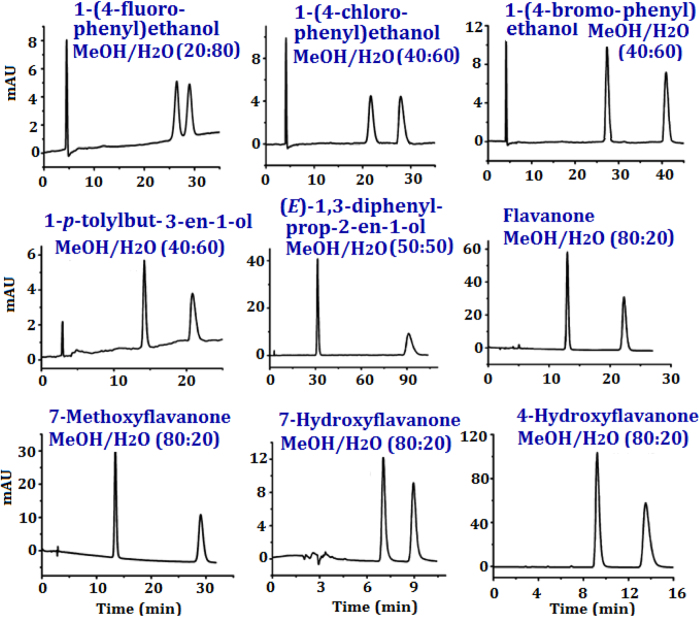
The representative enantioseparation chromatograms of selected analytes.

The peak resolutions of flavanone and methoxyflavanone with CCC3M4-CSP reached over 10. And the *R*_*s*_ values of all flavanoids with CCC3M4-CSP are about 4 ~ 5 times higher than those obtained with ealierly reported native and unfuctionalized perphenylcarbamated CD clicked CSPs^18^,^20^,^21^,^12^. And the *R*_*s*_ values are much higher than those in literature obtained with amylose tris(5-chloro-2-methylphenylcarbamate^32^, Chiralcel OD-H and Chiralpak AS-H CSPs^33^. Importantly, when we compare the enantioseparation results for flavanone, methoxyflavanones and hydroxylflavanones, one can find that the increased hydrophobicity with methoxy group resulted in higher resolution, while the increased hydrophility (with hydroxyl group) deteriorated the resolution. Similar behaviour was also found for hesperetin and naringenin, which presented lower chiral resolutions with the presence of hydroxy groups. The versatile enantioseparation of flavanoids with CCC3M4-CSP was evaluated at different mobile phases. The enantioseparations of flavanone with 1.0 mL/min acetonitrile/water (80/20) still achieved a *R*_*s*_ of 3.0, though it was rather lower than that with MeOH/H_2_O (80/20) (*R*_*s*_ = 11.2). The use of acetonitrile however led to shorter retention time than MeOH, due to the lower polarity of acetonitrile. The good resolution ability towards flavanoids can be explained with their interactions including inclusion complexation, dipole-dipole and hydrophobic interaction. The representative chromatograms for nine selected racemates are present in Fig. 4.

**Figure 5:**
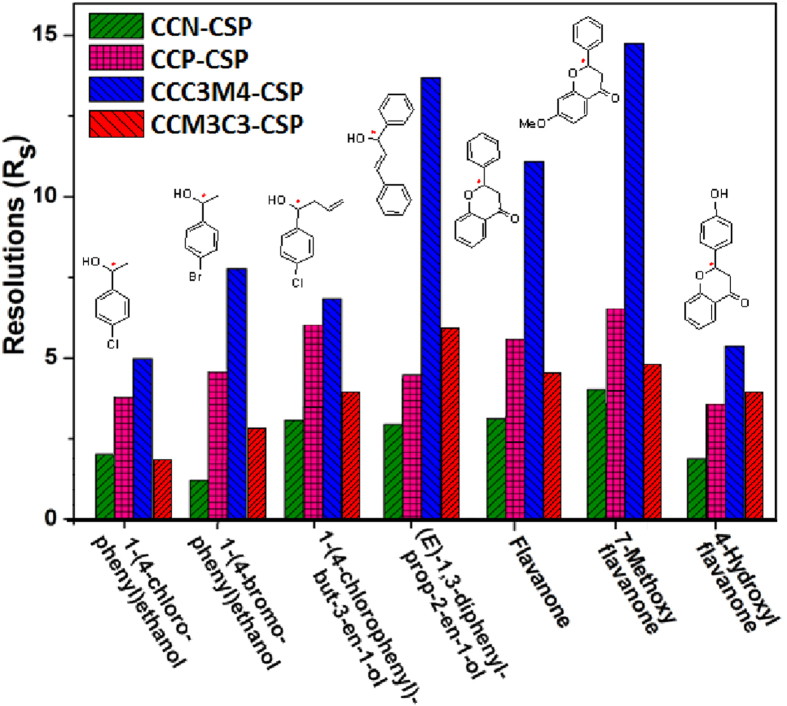
Comparison of chiral resolutions of 7 racemates with CCN-CSP and three phenylcarbamated CD clicked CSPs at the optimized conditions as in Table 1.

A vivid comparision of the functionality enhanced enantioselectivity of phenylcarbamated CD clicked CSPs (CCP-CSP^12^,^21^, CCC3M4-CSP and CCM3C4-CSP) towards seven aryl alcohols and flavanoids over native CD clicked CSPs (i.e., CCN-CSP^20^,^23^) is shown in Fig. 5. The improved enantioselectivity may be attributed to the introduction of phenylcarbamoyl groups on CD in constructing extra driving forces like π–π interactions, hydrogen bonding, and steric repulsion interactions besides inclusion complexation to promote the chiral recognition process^1^. Impressively, CCC3M4-CSP could afford over two-folds highers *R*_*s*_ for (*E*)-1,3-diphenylprop-2-en-1-ol, flavanone and 7-methoxyflavanone than all CD clicked CSPs in literature. However, CCM3C4-CSP exhibited lower resolution capabilities than CCP-CSP except 4-hydroxyl flavanone. This phenomenon revealed the functionalities of phenylcarbamoyl groups have to be carefully optimized to maximize the stability differences between the two inclusion complexes between functionalized CD and a pair of enantiomers. Similar behaviour of substituents position-tuned enantioselectivities was earlier observed for chloromethylphenylcarbarmated cellulose CSPs^30^, where the *meta-* and *para*-disubstituted derivatives were found to show higher chiral recognition than *ortho-* and *meta-* or *para*-disubstituted ones. The reason was explained with more acidic N-H protons of *meta-* and *para*-disubstituted phenylcarbamate celluloses would interact more strongly with appropriate enantiomers via hydrogen-bonding. Among two *meta-* and *para*-disubstituted derivatives, 3-Cl-4-CH_3_- functionalized downshifted the chemical shifts of N-H protons even further (more acidic) than 4-Cl-3-CH_3_- counterpart. This may explain the higher enantioselectivities of CCC3M4-CSP than CCM3C4-CSP in our CD cases.

**Figure 6:**
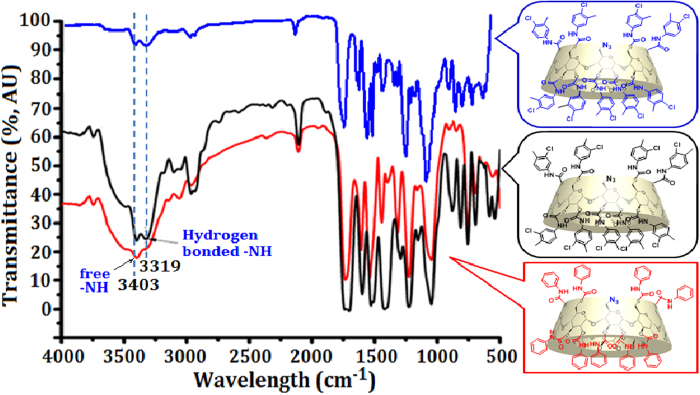
FT-IR spectra of mono-6^A^-azido-per(4-chloro-3-methyl)phenylcarbamate β-CD, mono-6^A^-azido-per(3-chloro-4-methyl)phenylcarbamate β-CD and mono-6^A^-azido-per(phenylcarbamate) β-CD. The inset showing the formation of intramolecular H-bonding between adjacent phenylcarbamate groups on neighbouring glucose units.

The improvement in chiral recognition ability of phenylcarbamated CD clicked CSPs over CCN-CSP with the introduction of phenylcarbamoyl group on CD rims may be explained with FT-IR analysis. As shown in Fig. 6, all three phenylcarbamated β-CD derivatives exhibit two identical peaks at 3403 and 3319 cm^−1^ in FT-IR spectra, which was assigned to free NH for the former and hydrogen-bonded NH for the latter. The hydrogen-bonded NH is not only involved in intramolecular H-bonding between adjacent phenylcarbamate groups on neighbouring glucose units, leading to a more stable and regular higher order structure of CD CSPs, but also form H-bonding interaction with racemates. These both functions contribute to the improvement of the chiral recognition ability of perphenylcarbamated CD CSPs in comparision to native CD CSPs. For CCC3M4-CSP, the introdution of an electron-donating 4-methyl group and an electron-withdrawing 3-chloro substituent on phenyl ring will help to promote the configuration-dependent inclusion for maximum stability difference between two complexes.

### Theoretical modeling

**Figure 7:**
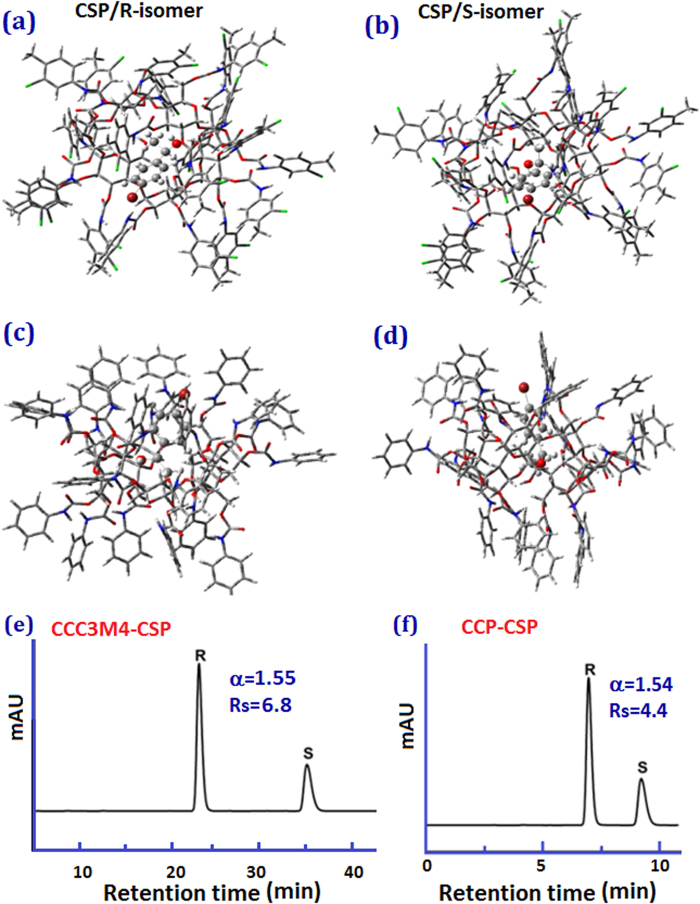
Optimized structure of (**a**) CCC3M4-CSP complex with *R-*1-(4-bromophenyl)ethanol, (**b**) CCC3M4-CSP complex with *S-*1-(4-bromophenyl)ethanol, (**c**) CCP-CSP complex with *R-*1-(4-bromophenyl)ethanol, and (**d**) CCP-CSP complex with *S-*1-(4-bromophenyl)ethanol in MeOH/H_2_O (50:50), Elution order of 1-(4-bromophenyl)ethanol enantiomers with (**e**) CCC3M4-CSP and (f) CCP-CSP at 1.0 mL/min MeOH/H_2_O (50/50).

To further elucidate the enhanced enantioselectivity for CCC3M4-CSP over CCP-CSP with 3- and 4-fuctionalities on phenyl group, quantum mechanics calculation was employed to evaluate the intermolecular interactions between CD chiral selector and enantiomers. We herein selected 1-(4-bromophenyl)ethanol as model analyte. Fig. 7a–d depicts the PM3 optimized structure of the complex for each enantiomer with CCC3M4-CSP and CCP-CSP. The interaction energy of the complexes was calculated using *E*_interaction_ = *E*_complex_-*E*_free-CD_-*E*_free-analytes_, where E is the total energy. The more negative the interaction energy, the stronger the interaction between (*R/S*)-1-(4-bromophenyl)ethanol and CSP, and the more stable is the inclusion complex.

The interaction energy of CCC3M4-CSP complex with (*R*/*S*)-1-(4-bromophenyl)ethanol was calculated to be –9.41 and –11.92 kcal/mol, respectively. And the interaction energy for CCP-CSP/(*R*)-1-(4-bromophenyl)ethanol and CCP-CSP/(*S*)-1-(4-bromophenyl)ethanol complex was calculated to be –12.13 and –12.47 kcal/mol, respectively. The more negative interaction energies of two CD clicked CSPs complexs with *S*-enantiomer of 1-(4-bromophenyl)ethanol suggest that S-enantiomer interacts more strongly with two CSPs than the *R*-enantiomer. As a result, the *R*- enantiomer should be eluted faster than *S*-enantiomer, which is in good agreement with experiment (Fig. 6e,f).

As discussed by Tipiol^34^, the difference in the interaction energies between CSP and two enantiomers reflects the energetic contribution to enantioselectivity. The interaction energies difference between CCC3M4-CSP/*R*-1-(4-bromophenyl)ethanol and CCC3M4-CSP/*S*-1-(4-bromophenyl)ethanol complex was calculated to be 2.51 kcal/mol, while it is only 0.34 kcal/mol for the complexes of CCP-CSP with (*R*)- and (*S*)-1-(4-bromophenyl)ethanol. The dramatically high interaction energy difference between the complexes of CCC3M4-CSP with a pair of enantiomers endows stronger driving force for chiral resolution. Hence, CCC3M4-CSP demonstrated significantly enhanced enantioselectivities than native CD and perphenylcarbamated CD based CSPs.

## Discussion

We have presented a general methodology to improve the enantioselectivity of CD clicked stationary phases via rational materials design. The new per(3-chloro-4-methyl)phenylcarbamated cyclodextrin clicked CSP featuring high CD immobilization and column efficiency exhibits high robustness in terms of sample loading and analysis repeatability. Chiral resolutions over 10 have been readily achieved for aryl alcohols and flavanoids. Experimental and simulation results revealed that the intrinsic chiral recognition and separation was attributed to the high interactions energy difference between the complexes of CD and enantiomers. Such highly enantioselective separation, synergizing the latest development in click chemistry and achievement in chromatography technology, has not been reported to date. This work may bring advanced materials science and chromatography technology to the forefront of practical application and a major societal need.

## Methods

### Materials and gernal procedures

**Figure 8:**
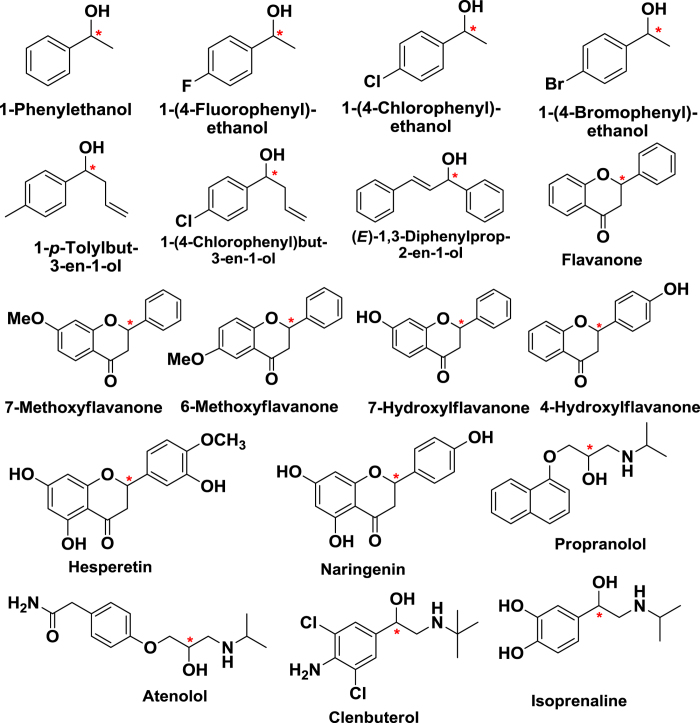
Structures of selected model analytes.

All reagents and solvents used in this study are commercially available and used without further purification. All racemates (structures shown in Fig. 8) and single enantiomes were purchased from Meryer Co. Ltd. (Shanghai, China). Kromasil spherical silica gel (5 μm, 100 Å) was purchased from Eka Chemicals (Bohus, Sweden). Deionized water was purified by Milli-Q system (Millipore, Bedford, MA, USA).

NMR spectra were collected on a Bruker AVANCE 500 MHz (Bruker Daltonics, Bremen, Germany; 500 MHz). Solid state ^13^C NMR was performed on a Avance III WB 400 MHz spectrometer (400 MHz, 9.4 T). Fourier-transform infrared spectra (FTIR) were collected on an ImPact410 FT-IR (Nicole, USA). Elemental analysis was performed on a Yanagimoto MT3CHN recorder.

### Synthesis of (3-chloro-4-methyl)phenylcarbamated CD (2a) and CCC3M4-CSP

Towards a freshly prepared mono-6^A^-azido-β-CD **1**^28^ (3.8 g, 3.2 mmol) solution in dry pyridine (25 mL), was added 3-chloro-4-methylphenyl isocyanate (18.36 g, 109.6 mmol) under the protection of nitrogen. The mixture was stirred at 90 ^o^C for 12 h. After cooling to room temperature, pyridine and un-reacted (3-chloro-4-methyl)phenyl isocyanate was removed with vacuum distillation. The residue dissolved in ethyl acetate (100 mL) was washed with water (3 × 50 mL). After evaporation of organic solvent, the residue was subjected to silica column chromatography with hexane/ethyl acetate (2:1, v/v) as eluent to afford the target product as a white solid (5.57 g, 41.9% yield). ^1^H NMR (500 MHz DMSO-d_6_) δ: 9.76-9.42 (m, NH, 20H), 7.50-7.26 (m, 2H-Ar, 20H), 7.25-7.11 (m, 6H-Ar, 20H), 7.00-6.70 (m, 5H-Ar, 20H), 5.56-5.22 (m, H-1, 21H), 4.82 (m, H-6, 14H), 4.30-3.93 (m, H-2, H-3, H-4 and H-5, 28H), 2.26-2.14 (m, C6-Ar-CH_3_,18H), 2.04-1.83 (m, C2-Ar-CH_3_ and C3-Ar-CH_3_, 42H); ^13^C NMR (125 MHz DMSO-d_6_) δ: 153.46, 152.78, 138.49, 137.83, 137.10, 133.57, 133.06, 131.52, 130.90, 130.76, 129.50, 129.35, 119.46, 119.16, 118.80, 118.54, 117.68, 117.44, 99.13, 78.20, 71.71, 69.93, 63.56, 19.19, 18.87; ESI-MS (*m/z*): 4467.65 (calcd.) and 4467.58 (found) for [M+Na^+^].

To a suspension of alkynyl functionalized silica **3**^22^ (4 g) in DMF (40 mL), was added mono-6^A^-azido-per(3-chloro-4-methyl)phenylcarbamated β-CD **2a** (4 g) and CuI(PPh_3_) (0.1 g). The reaction mixture was stirred at 85 ^o^C for 2 d. After filtration and washing with DMF, the residue was Soxhlet extracted with acetone for 24 h before vaccum dried to afford the *title* CCC3M4-CSP.

### Synthesis of (4-chloro-3-methyl)phenylcarbamated CD (2b) and CCM3C4-CSP

By adopting the same procedure for **2a**, 4-chloro-3-methylphenyl isocyanate (18.36 g, 109.6 mmol) was used to afford (4-chloro-3-methyl)phenylcarbamated CD **2b** as a white solid (52.7% yield). ^1^H NMR (500 MHz DMSO-d_6_) δ: 9.80-9.46 (m, NH, 20H),7.48-7.24 (m, 2H-Ar, 20H), 7.23-7.05 (m, 6H-Ar, 20H), 7.05-6.84 (m, 5H-Ar, 20H), 5.54-5.20 (m, H-1, 21H), 4.84 (m, H-6, 14H), 4.34-3.95 (m, H-2, H-3, H-4 and H-5, 28H); 2.25-2.16 (m, C6-Ar-CH_3_,18H), 2.05-1.86 (m, C2-Ar-CH_3_ and C3-Ar-CH_3_, 42H); ^13^C NMR (125 MHz DMSO-d_6_) δ: 153.53, 152.75, 138.21, 137.56, 136.88, 136.00, 135.28, 129.38, 128.69, 127.26, 126.99, 122.01, 121.52, 121.19, 119.14, 118.03, 99.38, 78.16, 71.86, 70.05, 63.61, 20.18, 19.86; ESI-MS (*m/z*): 4467.65 (calcd.) and 4467.59 (found) for [M+Na^+^].

By adopting the similar procedure for CCC3M4-CSP, the click chemistry between **2b** and alkynyl functionalized silica under the catalysis of CuI(PPh_3_) afforded the target CCM3C4-CSP.

### Column packing and HPLC experiments

The CD clicked CSP was packed into columns by the conventional slurry method, where the CSP suspension in methanol was packed into stainless steel columns (4.6 × 250 mm) under constant pressure (9000 psi) using packing system (Lab Alliance Scientific). The packing pressure was maintained for at least 0.5 h. The column was rinsed and equilibrated with the mobile phase before use.

All HPLC experiments were performed on an Agilent 1260 HPLC system, which is comprised of a G1322A degasser, G1315D diode array detection system, G1329B quaternary pump, G1331C auto-injector and a G1316A temperature controller and Agilent Chem Station data manager software (Agilent Technologies, Palo Alto, CA, USA). All chromatograms were collected at 254 nm detection at 25 ^o^C with a 1.0 mL/min flow rate of mobile phase. All analyte solutions in methanol (100 μg/mL concentration) were injected to column with a volume of 20 μL for HPLC. The retention factor k_n_ were calculated with the formula: 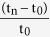, where t_0_ was the elution time of solvent peak. The selectivity factor (α) was calculated using k_2_/k_1_, meanwhile, peak resolution (*R*_*s*_) was evaluated using the formula: 1.18 × (t_2_−t_1_)/(w_1_+w_2_), where w_1_ and w_2_ were the half-peak width of the corresponding enantiomer, respectively.

### Molecular modeling

The complexes of CD CSP and enantiomers in reversed-phase HPLC conditions are selected for the simulation of optimized interaction structures. The energy-minimized structures were firstly obtained with the chiral carbon pointing towards the narrow rim of CD during the docking process using PM3 method^35^. During docking, the enantiomer was rotated ~30^o^ every time in CD cavity. The optimization was carried out after each rotation till the stable structure of CD/enantiomer complex was found. Based on the optimized structures, the interaction energy between CD-CSP complex and enantiomers was calculated.

## Additional Information

**How to cite this article**: Tang, J. *et al.* Engineering Cyclodextrin Clicked Chiral Stationary Phase for High-Efficiency Enantiomer Separation. *Sci. Rep.* **5**, 11523; doi: 10.1038/srep11523 (2015).

## Figures and Tables

**Figure 1:**
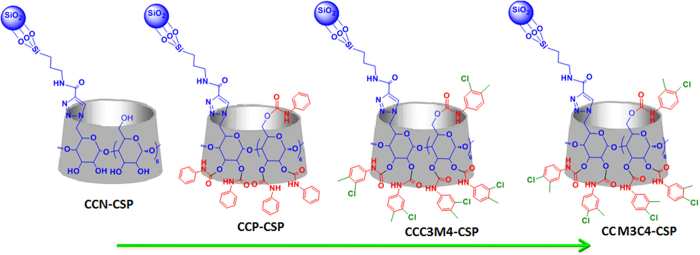
Structure of CD, perphenylcarbamated CD, per(3-chloro-4-methyl)phenylcarbamated CD and per(4-chloro-3-methyl)phenylcarbamated CD clicked CSPs (denoted as CCN-CSP, CCP-CSP, CCC3M4-CSP and CCM3C4-CSP, respectively).

**Table 1:**
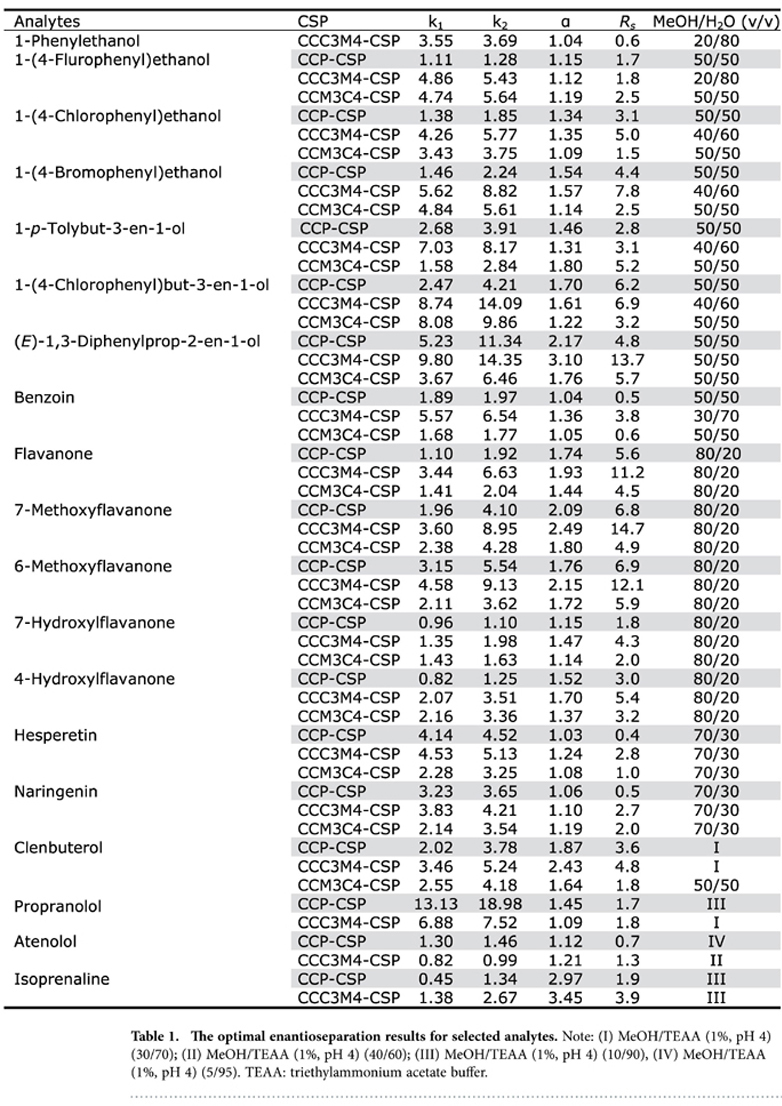
The optimal enantioseparation results for selected analytes.

Note: (I) MeOH/TEAA (1%, pH 4) (30/70); (II) MeOH/TEAA (1%, pH 4) (40/60); (III) MeOH/TEAA (1%, pH 4) (10/90), (IV) MeOH/TEAA (1%, pH 4) (5/95). TEAA: triethylammonium acetate buffer.
